# Boredom is the root of all evil—or is it? A psychometric network approach to individual differences in behavioural responses to boredom

**DOI:** 10.1098/rsos.211998

**Published:** 2022-09-28

**Authors:** Maik Bieleke, Leonie Ripper, Julia Schüler, Wanja Wolff

**Affiliations:** ^1^ Department of Sport Science, University of Konstanz, 78457 Konstanz, Germany; ^2^ Department of Educational Psychology, University of Bern, 3012 Bern, Switzerland

**Keywords:** boredom proneness, functional theories of emotion, adaptive and maladaptive behaviour, psychometric network modelling, boredom avoidance and escape, dealing with boredom

## Abstract

Functional accounts of boredom propose that boredom serves as an impartial signal to change something about the current situation, which should give rise to adaptive and maladaptive behaviour alike. This seemingly contrasts with research on boredom proneness, which has overwhelmingly shown associations with maladaptive behaviour. To shed light on this discrepancy, we disentangled boredom proneness from individual differences in (i) the *urge to avoid and escape boredom* and (ii) *adaptive and maladaptive ways of dealing with boredom* by developing corresponding trait scales. In a study with *N* = 636 participants, psychometric network modelling revealed tight associations between boredom proneness and less adaptive and (especially) more maladaptive ways of dealing with boredom. However, its associations with the urge to avoid and escape boredom were rather weak. Importantly, a higher urge to avoid and escape boredom was linked not only to more maladaptive but also to more adaptive ways of dealing with boredom. This pattern of results was robust across various specific behaviours that have previously been linked to boredom. Our findings provide novel evidence for functional accounts of boredom from an individual difference perspective, cautioning against a shallow view of boredom as being associated with purely maladaptive behaviour.

## 1. Introduction

Boredom is a complex construct that most commonly refers to an aversive, transitory state people experience in some situations (state boredom) or to a general inclination to experience boredom frequently and across different situations (trait boredom).^1^ As a transitory state, boredom is a ubiquitous sensation that people can experience in various contexts [2]: when studying for school or working in the office, waiting for a connection at the train station or for an appointment at a medical facility, being alone and having nothing particular to do, and so on [3]. In all of these contexts, boredom arises when individuals perceive their situation as unsatisfying and would like to pursue more rewarding activities instead. In turn, being bored is an inherently unpleasant experience [4]. Importantly, by being such an aversive sensation that arises when individuals fail to engage with what they are currently doing, boredom acts as a strong catalyst to do something else instead. Put in more technical language, boredom functions as a signal for exploration [5,6] by simultaneously increasing general reward sensitivity [7] and downweighing the value of an ongoing activity [8]. This motivating property of boredom is acknowledged in current theorizing about of boredom's function as an impartial signal for change in goal-directed behaviour (e.g. [9,10]). However, empirical research that looks into stable differences in boredom proneness tends to find that boredom is associated with maladaptive consequences (e.g. [11,12])—in line with Søren Kierkegaard’s [13] famous notion of boredom as ‘a root of all evil'. This is at odds with the theoretical proposition of boredom as being rather impartial to the change it prompts, and therefore potentially causing adaptive and maladaptive behaviours alike (e.g. [14,15]). Here, adaptivity refers to behaviours that benefit the individual in a Darwinian sense (e.g. prolonged life expectancy) and that are encouraged by the society as a whole (e.g. in terms of societal norms).

### 1.1. Functional accounts of boredom versus research on boredom proneness

From the perspective of such *functional accounts of boredom* [14,16]), boredom serves as a signal that it might be better to let go of the ongoing activity and engage in potentially more rewarding activities instead. This notion of a functional signal has been expanded by some authors who propose that boredom is an inherently adaptive sensation (e.g. [17–19]). Corroborating this line of thinking, neuroscientific research shows that boredom increases the sensitivity for rewards [7], which in turn leads to a devaluation of the current activity relative to other, potentially more rewarding activities [8]. In computational and empirical work, this notion has been reinforced by showing that boredom is an important driver of explorative behaviour [20,21]. Importantly, this signal is assumed to be impartial in the sense that boredom does not imply *how* this change should be brought about. Instead, characteristics of the individual as well as of the current situation determine which behaviour is changed to in response to boredom. Essential is that the change leads to an experience that is sufficiently different from the ongoing activity. For instance, in a series of experiments by Bench & Lench [22], people preferred to see pleasant pictures after having viewed a series of unpleasant pictures for several minutes; however, they also preferred to see unpleasant pictures after having viewed a series of pleasant pictures. This reversal of preferences is in line with predictions made by functional accounts and the assumed impartiality regarding the direction of behaviour prompted by the experience of boredom. Taken together, functional accounts of boredom predict that boredom should be associated with more adaptive and more maladaptive behaviour alike.

However, this prediction seems to be at odds with a large body of literature that has investigated the consequences of *boredom proneness*, which is defined as the ‘tendency toward experiencing boredom' ([11, p. 5]. People prone to experience boredom are bored more often, experience boredom more intensely, and perceive their life as more boring overall [23]. Importantly, boredom proneness has consistently been linked to lower levels of adaptive behaviour and higher levels of maladaptive behaviour (reviews in [12,24]). People high in boredom proneness engage in unhealthy behaviours like emotional eating [25], smoking [26], drug-taking [27] and alcohol consumption [28], but refrain from healthy behaviours like physical activity [29] or complying with social distancing guidelines during the COVID-19 pandemic [30,31], exposing them to a greater health-related risks (e.g. to suffer death from cardiovascular disease, [32]). Boredom proneness is further associated with poor psychosocial development [33], high aggression and hostility [34,35], and social withdrawal [33,36,37]. Also, boredom proneness is linked to more maladaptive (e.g. excessive use of smartphones, watching more TV and movies, [2,38,39] and less adaptive leisure time activities (e.g. sports, reading of books and newspapers, [2,29,38]). Finally, boredom proneness is involved in risk behaviour [40,41], which might underlie preferences for unsafe driving [42,43], inclinations toward gambling [44–46] and a higher probability of violating social rules [31]. In sum, individual differences in boredom proneness are consistently associated with engaging less in adaptive behaviours and more in maladaptive behaviours.

### 1.2. Disentangling boredom proneness from individual differences in tendencies to avoid, escape from and deal with boredom

Taken together, functional accounts assume that boredom should steer more adaptive and maladaptive behaviours alike; however, research on boredom proneness has overwhelmingly shown associations with more maladaptive and less adaptive behaviour. What could be the reason for this apparent discrepancy? First, functional accounts revolve around the core assumption that boredom creates an *urge to avoid or escape boredom*. Some authors have likened the function of boredom for goal-directed behaviour to the function of pain as an essential driver of behaviour change [19,47]. According to this view, the intensity of the boredom signal should indicate how urgently a change of the current situation is needed. This idea, however, is not captured by boredom proneness as it is currently conceptualized. Boredom proneness refers to the failure to engage in a life that is not boring (as reflected in items like ‘I often find myself at "loose ends", not knowing what to do’ and ‘Much of the time, I just sit around doing nothing', [11]) but remains agnostic to the urge to avoid or escape boredom: people high in boredom proneness could experience an urge to avoid and escape boredom that they struggle to satisfy. However, they might also entirely lack this urge because they are less sensitive to boredom and tolerate high levels of boredom. Indeed, people differ in their tolerance for pain [48] and other aversive experiences like emotional distress [49]. Analogous to pain and distress, boredom is an aversive signal that people try to avoid, and some individuals might be more sensitive to this signal (at a given signal strength) and thus more likely to avoid or escape it. Accordingly, one reason for the apparent discrepancy between predictions derived from functional accounts of boredom and empirical findings from research on boredom proneness could be that the current conceptualization of boredom proneness does not capture individual differences in the urge to avoid and escape boredom.

Second, testing the predictions made by functional accounts of boredom requires that people can decide whether to *deal with boredom in an adaptive or in a maladaptive way*. Such a situation is relatively easy to create in laboratory studies examining how people respond to acute episodes of state boredom (e.g. [22]). However, in daily life people might be restricted in the ways in which they can deal with boredom by external circumstances (e.g. in school most ways of dealing with boredom in class are maladaptive, such as daydreaming; other examples include prison life, hospital stays and global pandemics) and internal factors. Attesting to the latter, it is important to note that boredom proneness consistently displays strong negative associations with self-control (e.g. [50]), meaning that people who are prone to experience boredom are also likely to have poor self-control. In addition, theoretical work points towards an intrinsic link between the experience of boredom and the need to apply self-control in order to deal with being bored [5,10]. In the same vein, previous studies have reported substantial negative correlations between −0.61 and −0.74 [29,31,51], meaning that measures of boredom proneness and self-control share between 35 and 55% of their variance. Critically, low self-control has been consistently associated with engaging in more maladaptive behaviours and in less adaptive behaviours [52–54], closely mirroring research on the consequences of boredom proneness. Accordingly, another reason for the discrepancy between predictions made by functional accounts of boredom and research on boredom proneness might pertain to the confound between boredom proneness and poor self-control.

## 2. Present research

While there is increasing consensus about the important role of state boredom as a motivator of goal-directed behaviour, research suggests that boredom-prone individuals engage more in maladaptive behaviours and less in adaptive behaviours. Not only is the existence of a trait with solely negative consequences implausible from an evolutionary perspective, the consistent pattern of findings also contradicts predictions that can be derived from functional accounts of boredom. The apparent discrepancy between empirical research and functional accounts might reflect that current measures of boredom proneness neither capture the urge to avoid and escape boredom (i.e. an individual's sensitivity to the sensation) nor the ways in which people deal with boredom. Here, we developed measures tapping into individual differences regarding these two essential aspects to disentangle them from measures of boredom proneness. We expected a stronger urge to avoid and escape boredom to be associated with both more adaptive and more maladaptive ways of dealing with boredom and the corresponding behaviours. These predictions reflect the idea that boredom is an inherently impartial signal that motivates change irrespective of whether the associated behaviour is adaptive or maladaptive. By contrast, we expected the urge to avoid and escape boredom to display weak (if any) associations with boredom proneness, as individuals high in boredom proneness could feel the urge to avoid and escape boredom (but fail to do so) or they could lack such an urge (tolerating or giving in to boredom). Finally, previous research suggests that boredom proneness should be associated positively with maladaptive ways of dealing with boredom and negatively with adaptive ways of dealing with boredom.

## 3. Methods

### 3.1. Participants

We recruited participants via Amazon Mturk and CrowdResearch (requirements: greater than or equal to 90% approval rate, greater than or equal to 100 HITs) who received $1 as compensation. To ensure high data quality, we used the CrowdResearch option to block workers with a history of low-quality work, a captcha to guard against bots, and an instructional and a self-report attention check. We followed the recommendation to recruit as many participants as possible with our available resources to obtain the most stable estimates [55]. A total of 683 participants were recruited, of which 43 did not complete the study. Of the remaining 640 participants, we excluded four who failed the attention check. The final sample size was *N* = 636 (age: *M* = 39.9 years, s.d. = 12.5, 45.4% female). This sample size meets the recommendations for the various statistical procedures we applied. For instance, it substantially exceeds the threshold for obtaining robust estimates of correlation coefficients [56], provides high power to establish the significance of even small effects in multiple regression analyses [57], and allows us to discover true networks in psychometric network analyses in a replicable manner [58]. Most participants reported working full-time (61.6%) or being self-employed (14.9%), and a majority indicated holding a college degree (82.5%). About half of the sample (47.4%) reported an income between $20 000 and $59 999.

### 3.2. Instruments

We developed custom scales to measure individual differences in *boredom avoidance and escape* (BAE) and in adaptive and maladaptive ways of *dealing with boredom* (DWB). Moreover, we assessed several specific adaptive and maladaptive behaviours that have been linked to boredom proneness in prior research. All materials are available in the OSF (https://osf.io/nbw95).

#### 3.2.1. Boredom avoidance and escape scale

The development of this scale was based on the idea that people differ in their tendency to avoid boredom and escape from it. As a starting point, we used the Regulation subscale of the Distress Tolerance Scale [49]. Distress tolerance is defined as ‘the capacity to experience and withstand negative psychological states' ([49, p. 83], which fits well to the notion of boredom as an aversive signal. Distress regulation refers to ‘efforts to avoid negative emotions and using rapid means of alleviating the negative emotions' ([49, p. 84], and thus taps into behavioural aspects of avoiding and escaping aversive psychological states. This made the subscale ideally suited for addressing our research questions. We adapted its three items to the context of boredom and constructed several additional items reflecting the avoidance of or the escape from boring situations, activities, places and individuals—features of the context known to be relevant for the experience of boredom [3]. This resulted in a preliminary set of 17 items (table 1) to which participants responded on 7-point Likert scales (1 = *strongly disagree*, 7 = *strongly agree*).

**Table 1.** Items measuring boredom avoidance and escape (BAE) tendencies.

no.	item
*1*	*I'll do anything to avoid feeling bored*.*^a^*
2	I make an effort to avoid getting bored.
3	I try to avoid doing things that could bore me.
4	I actively try to avoid boredom.
*5*	*I do all I can to avoid getting into boring situations.*
6	I make an effort to avoid situations where I might get bored.
7	I try hard to avoid boring places.
8	I stay away from boring people.
*9*	*I'll do anything to stop feeling bored***.***^a^*
*10*	*When I feel bored, I must do something about it immediately.^a^*
11	When I get bored, I try to do something else as soon as possible.
12	When boredom arises, I immediately look for something else to engage in.
13	I try to get a boring activity over with as soon as possible.
14	When I am bored, I have a strong urge to change something.
15	I try to leave boring situations immediately.
16	I leave boring places as soon as possible.
17	When people bore me, I try to leave.

*Note.* Items marked with an *^a^* were adapted from the Distress Tolerance Scale [49]. Items in italic font were selected for the final BAE scale (details in the main text).

#### 3.2.2. Dealing with boredom scale

The development of this scale was based on the assumption that people can deal with boredom in both adaptive and maladaptive ways. We constructed a preliminary set of items that corresponded to the conceptualization of adaptivity that we have adopted here (i.e. benefitting the individual fitness and encouragement by the society) and that reflected adaptive ways of dealing with boredom (seven items) versus maladaptive ways of dealing with boredom (three items; table 2). All items started with the phrase ‘When I am bored…’ and participants indicated their agreement with each statement on a 7-point Likert scale (1 = *strongly disagree*, 7 = *strongly agree*).

**Table 2.** Items measuring adaptive and maladaptive ways of dealing with boredom (DWB).

no.	item
	When I am bored…
1	…I do things that are good for me.
2	*…I do things that are generally known to be bad.*
3	…I do things other people would advise me to do.
4	*…I do things that bring benefits in the long run.*
5	…I do something reasonable.
6	*…I use my time doing something useful.*
7	*…I do nonsense.*
8	*…I try to be productive.*
9	*…I do things that are fun, even if I might regret it later.*
10	…I turn my attention to important tasks that I have to do.

*Note.* Items in italic font were selected for the final DWB scale (details in the main text).

#### 3.2.3. List of specific adaptive and maladaptive behaviours

We identified several adaptive and maladaptive behaviours that have been associated with boredom proneness in previous research. Each behaviour was assessed with a single item (table 3). The items pertaining to physical activity [59], problematic eating [60], drug-taking [61] and self-inflicted injury [62] are based on existing measures, the remaining items were developed for the present study. For each item, participants indicated how often they typically show the specified behaviour in general (i.e. not only when being bored, 1 = *never*, 6 = *very frequently*). Note that these items are not meant to form a scale; rather, each item measures a specific behaviour.

**Table 3.** Items measuring specific adaptive and maladaptive behaviours.

domain	item
*adaptive behaviours*	
physical activity	engaging in physical activity long enough to work up a sweat (e.g. taking stairs, doing sports)
fulfillment of duties	fulfilling your duties regarding work/university/school/household
prosocial behaviour	being friendly towards others
social relationships	contacting your friends or family
reading	reading a book/newspaper/article
*maladaptive behaviours*	
problematic eating	eating extremely large amounts of food at one time and feeling that your eating was out of control at that time
smoking	smoking (e.g. cigarettes, a pipe, cigars)
drug-taking	using illegal drugs or prescription medication for non-medical reasons
alcohol consumption	drinking an amount of alcohol that you later regret
self-inflicted injury	harming or hurting your body on purpose (for example, cutting or burning your skin, hitting yourself, or pulling out your hair)
aggression/hostility	behaving in a hostile or aggressive way
social withdrawal	avoiding other people
rule violations	doing things that are forbidden
excessive smartphone use	using your smartphone excessively (e.g. social media, gaming)
excessive media use	watching TV/movies/series excessively
general risk behaviour	putting yourself in risky, dangerous situations
pathological gambling	engaging in gambling
financial risk-taking	making imprudent financial decisions (e.g. risking money, lavishing money)
traffic risk-taking	taking risks in traffic (e.g. driving fast and recklessly)

#### 3.2.4. Boredom proneness

We measured boredom proneness with the *Short Boredom Proneness Scale* (SBPS, [63]), which comprises eight items (sample item: ‘I often find myself at loose ends, not knowing what to do').

#### 3.2.5. Demographic questionnaire

We assessed basic demographic information (age, gender) along with information about participants' objective socio-economic status (SES) in terms of their annual income, employment status, and education. Moreover, subjective SES was measured using a 7-step ladder on which participants placed themselves (1 = *least money, education, and respected jobs*, 7 = *most money, education, and respected jobs*, [64]).

### 3.3. Procedure

The study was conducted online using the online survey software Qualtrics. All materials are available in the OSF (https://osf.io/nbw95). Participants gave their informed consent and were then forwarded to an instructional manipulation check [65]. Afterward, participants worked on the questionnaires assessing boredom avoidance and escape (BAE) tendencies, adaptive and maladaptive ways of dealing with boredom and boredom proneness (SBPS). They then indicated the frequency of engaging in a list of adaptive and maladaptive behaviours. The study concluded with a demographic questionnaire and a self-report attention check, in which participants indicated whether they had worked seriously on the study (yes/no).

### 3.4. Data analysis

All analyses were conducted in the statistical environment R version 4.1.3 [66]. We relied primarily on the *tidyverse* version 1.3.1 [67] for data wrangling and plotting and *EGAnet* version 0.9.8 [68] for psychometric network analysis. The list of packages can be accessed via the OSF (https://osf.io/nbw95) along with the complete dataset and the analysis script. Our analysis involved three steps: (i) establishing the psychometric properties of the scales developed for measuring BAE and DWB, (ii) examining the nomological network of BAE, DWB and SBPS, and (iii) testing the predictive value of BAE, DWB and SBPS for specific adaptive and maladaptive behaviours.

#### 3.4.1. Psychometric properties of the boredom avoidance and escape, and dealing with boredom scales

To establish the psychometric properties of the BAE and the DWB scales, we conducted *exploratory graph analyses* (EGA, [69]). EGA is a recently developed psychometric tool for estimating networks and detecting communities in multivariate data. Unlike other approaches to characterizing the psychometric properties of psychological constructs (e.g. latent variable modelling), psychometric network models are not tied to a particular set of assumptions (e.g. latent variables cause correlations between items) but allow a more data-driven and unrestricted approach (for a more detailed discussion, see [70]). The EGA approach is recommended for the validation of trait questionnaires because it (i) outperforms traditional approaches in terms of accurately identifying latent structures and (ii) reduces researchers' degrees of freedom and thus the potential of errors and biases [71]. Here, we relied on the EGA() function to estimate separate Gaussian graphical models for both the BAE and the DWB scale using the graphical least absolute shrinkage and selection operator (GLASSO). These models comprise nodes (representing individual variables) and edges between them (representing conditional dependence). We set the min–max ratio for network sparsity (*λ*) to 0.1 and applied the extended Bayesian information criterion (EBIC) to select a model based on a hyperparameter (*γ*) of 0.5. The Walktrap algorithm was used to identify communities within both empirical networks.

Next, we selected the items that contributed best to the construct of interest based on an evaluation of the networks. These items were subjected to the bootEGA() function to run EGAs on each of 1000 bootstrapped samples drawn from a multivariate normal distribution. The resulting sampling distribution of EGA networks provides information about the ability of a network to replicate across samples (e.g. producing the same number of communities) and thus yields a ‘typical' network. The empirical networks were further subjected to confirmatory factor analysis (CFA) to investigate how well they fit the data. We relied on a maximum-likelihood estimation with robust standard errors and a Satorra–Bentler scaled test statistic (MLM). We report *χ*^2^, RMSEA, SRMR, CFI and TLI, considering values of less than or equal to 0.06 for RMSEA, less than or equal to 0.08 for SRMR and greater than or equal to 0.95 for CFI and TLI as indicating good fit [72]. Finally, the reliability of the resulting scales was assessed in terms of Cronbach's α and McDonald's ω, for which values greater than or equal to 0.70 are considered acceptable [73].

#### 3.4.2. Associations between boredom avoidance and escape, dealing with boredom and the Short Boredom Proneness Scale

We examined the associations between BAE, DWB and SBPS in terms of bivariate Pearson correlations between the aggregate scale scores and their confidence intervals. As a robustness check (e.g. to hedge against potential violations of distributional assumptions), we also report bootstrapped estimates of coefficients and confidence intervals. To further scrutinize these associations, we submitted all items simultaneously to an EGA, evaluated the estimated network, tested its stability with bootstrapping and investigated the typical network. We report common centrality indices of the empirical network (i.e. betweenness, closeness, strength).

#### 3.4.3. Explaining specific adaptive and maladaptive behaviour with boredom avoidance and escape, dealing with boredom and the Short Boredom Proneness Scale

We used the EGA approach to collapse the observed scores of the 19 specific adaptive and maladaptive behaviours into *network scores* using the net.scores() function. These network scores reflect each behaviour's strength within a community (i.e. including cross-loadings) and can be interpreted as item weight. This yields a reduced set of network scores for each participant that summarizes their behaviour in the most parsimonious way. We first regressed the network scores on SBPS and then added BAE and DWB. Scores on DWB items reflecting adaptive ways of dealing with boredom (DWB-A) were used as predictor of adaptive behaviours, whereas scores on DWB items reflecting maladaptive ways of dealing with boredom (DWB-M) were used as predictor of maladaptive behaviours. In the final model, we examined the individual contributions of all three scales. We report the (standardized) beta coefficients and their confidence interval, along with the explained variance of each model (*R*²). Moreover, we also report bootstrapped regression coefficients and confidence intervals as a robustness check (e.g. to hedge against potential violation of distributional assumptions).

## 4. Results

### 4.1. Psychometric properties of the boredom avoidance and escape, and dealing with boredom scales

#### 4.1.1. Boredom avoidance and escape scale

The initial EGA revealed three communities: one community comprised four items that reflected an urge to avoid (e.g. ‘I'll do anything to avoid boredom', Items 1 and 5) or escape boredom (e.g. ‘I'll do anything to stop feeling bored', Items 9 and 10), this included all three items adapted from the Distress Tolerance Scale. The other two communities comprised items reflecting a milder tendency to avoid or escape from boredom. Items in one community pertained to changing one's personal activities (e.g. ‘I try to avoid things that could bore me', Items 11, 12, 13 and 14) and items in the other community pertained to changing one's environment (‘When people bore me, I try to leave', Items 2, 3, 4, 6, 7, 8, 15, 16 and 17). As our focus was on measuring robust and strong urges to avoid and escape boredom analogous to the approach taken in the Distress Tolerance Scale, we retained only the four items that were assigned to the first community for the analyses.

**Figure 1.**
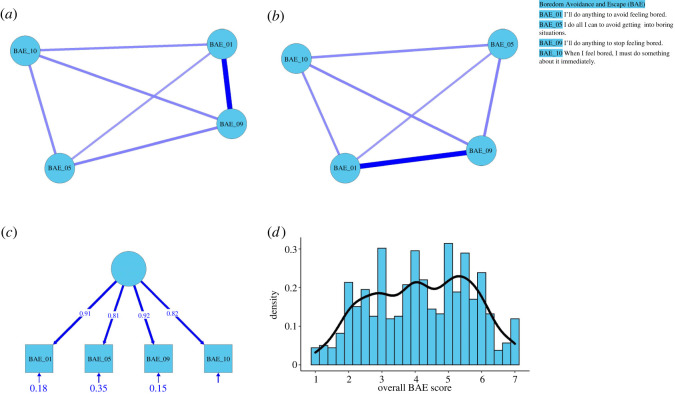
Psychometric properties of the boredom avoidance and escape (BAE) scale. *Note.* The empirical network (*a*) and the typical network (*b*) suggest that the BAE scale represents a unidimensional and replicable network of four items. Together with the results of a CFA (*c*), this suggests that responses to all four items can be averaged into a single BAE score (*d*).

We subjected these four items to a second EGA (figure 1*a*) and used bootstrapping to investigate the robustness of the resulting network. The empirical network turned out to be unidimensional and stable: across all 1000 bootstrapped samples, the solution was always unidimensional and comprised all four items. The typical network (figure 1*b*) corresponded closely to the empirical network. Moreover, the CFA revealed that the solution provided excellent fit to the data (figure 1*c*), *χ*^2^_(2)_ = 3.15, *p* = 0.206, RMSEA = 0.030, 90% CI [0.000, 0.077], *p* = 0.701, CFI = 0.999, TLI = 0.997, SRMR = 0.009. Finally, the reliability of the overall score (*M* = 4.17, s.d. = 1.50; figure 1*d*) was excellent as well (Cronbach's *α* = 0.92, 95% CI [0.91, 0.93], McDonald's *ω* = 0.92, 95% CI [0.91, 0.93]).

#### 4.1.2. Dealing with boredom scale

The initial EGA revealed two communities, one comprising the three maladaptive ways of dealing with boredom (Items 2, 7 and 9) and the other one comprising the seven adaptive ways (Items 1, 3, 4, 5, 6, 8 and 10). This finding cautioned against using the DWB scale as a unidimensional scale with reverse-coding. Accordingly, we created two separate subscales for adaptive and maladaptive ways of dealing with boredom (referred to as DWB-A and DWB-M, respectively). We used all of the available three maladaptive items for the DWB-M subscale as they were assigned to the same community. We added an equal number of adaptive items for the DWB-A subscale based on loadings in a complementary exploratory factor analysis (with principal axis method and oblimin rotation). The final DWB scale thus comprised six items in total, three items representing adaptive (Items 4, 6 and 8) and three items representing maladaptive (Items 2, 7 and 9) ways of dealing with boredom.

**Figure 2.**
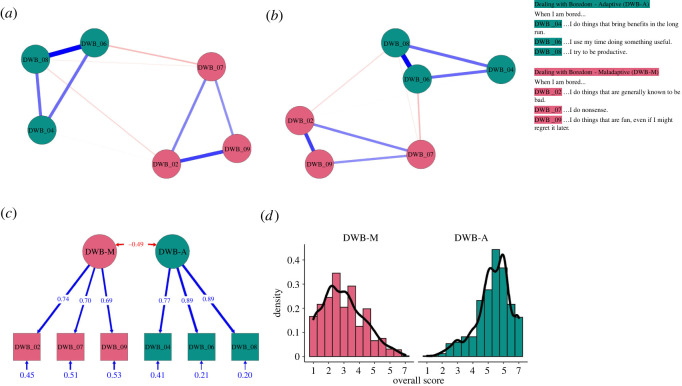
Psychometric properties of the dealing with boredom (DWB) scale. *Note.* The empirical network (*a*) and the typical network (*b*) suggest that the DWB scale represents a two-dimensional and replicable network of six items (i.e. three per subscale). Together with the results of a CFA (*c*), this suggests that responses to the six items can be averaged into separate DWB-A and DWB-M scores (*d*).

We then subjected these items to a second EGA, which confirmed the two communities (adaptive and maladaptive, figure 2*a*). Bootstrapping showed that this two-dimensional empirical network was stable as well: all items were assigned to their respective community across all 1000 iterations and the typical network (figure 2*b*) corresponded closely to the empirical network. Moreover, the CFA showed that the solution fit the data very well (figure 2*c*), *χ*^2^_(8)_ = 26.83, *p* = 0.001, RMSEA = 0.061, 90% CI [0.040, 0.083], *p* = 0.181, CFI = 0.984, TLI = 0.970, SRMR = 0.045. The overall scores (figure 2*d*) of the adaptive (*M* = 5.30, s.d. = 1.13, *α* = 0.89, 95% CI [0.87, 0.90], *ω* = 0.89, 95% CI [0.87, 0.90]) and the maladaptive (*M* = 2.99, s.d. = 1.34, *α* = 0.75, 95% CI [0.72, 0.78], *ω* = 0.75, 95% CI [0.72, 0.79]) DWB scales showed good and satisfactory reliability, respectively.

### 4.2. Nomological network of boredom avoidance and escape, dealing with boredom and the Short Boredom Proneness Scale

**Figure 3.**
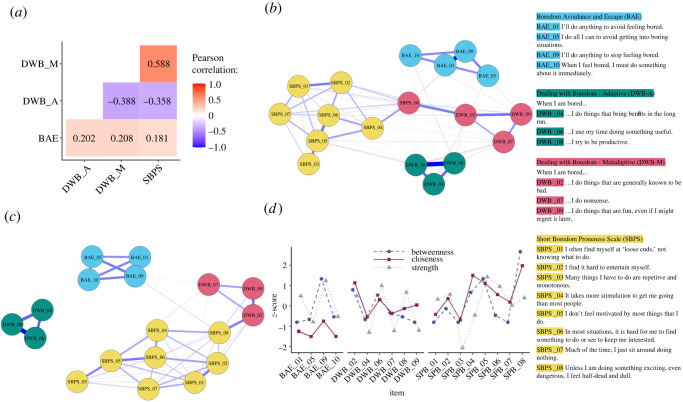
Nomological network of the BAE, DWB-A, DWB-M and SBPS. *Note*. Correlations between scales (*a*) are based on a sample of *N* = 636 and thus significant if *r* ≥ 0.077. The empirical network (*b*) and the typical network (*c*) suggest that the different scales tap into four separable aspects of boredom, although boredom proneness (SBPS) is linked to maladaptive ways of dealing with boredom (DWB-M) especially via Item 8 of the SBPS. This is also reflected in the centrality indices (*d*).

As can be seen in figure 3*a*, there was a small positive correlation between SBPS and BAE, *r* = 0.18, 95% [0.11, 0.26], *r*_boot_ = 0.14, 95% [0.05, 0.22], indicating that people with a greater tendency to escape and avoid boredom reported slightly more boredom proneness. Moreover, we observed a medium negative correlation between SBPS and DWB-A, *r* = −0.36, 95% [−0.42, −0.29], *r*_boot_ = −0.37, 95% [−0.45, −0.29], and a large positive correlation between SBPS and DWB-M, *r* = 0.59, 95% [0.53, 0.64], *r*_boot_ = 0.55, 95% [0.48, 0.61], suggesting that more boredom-prone individuals are inclined to deal with boredom in less adaptive and more maladaptive ways. By contrast, the BAE displayed small and positive correlations with both DWB-A, *r =* 0.20, 95% CI [0.13, 0.28], *r*_boot_ = 0.23, 95% CI [0.15, 0.31] and DWB-M, *r* = 0.21, 95% CI [0.13, 0.28], *r*_boot_ = 0.17, 95% CI [0.10, 0.26], indicating that the tendency to escape and avoid boredom is associated with more adaptive and more maladaptive ways of DWB. Finally, there was a negative correlation between DWB-A and DWB-M, *r* = −0.39, 95% CI [−0.45, −0.32], *r*_boot_ = −0.40, 95% CI [−0.47, −0.32].

Turning to the EGA, we observed the expected solution with four communities representing each of the four scales (i.e. BAE, DWB-A, DWB-M and SBPS; figure 3*b*). Item 8 of the SBPS scale ('Unless I am doing something exciting, even dangerous, I feel half-dead and dull') was assigned to the DWB-M community in the empirical network, and the bootstrap analysis suggests that this item was similarly likely to be assigned to the SBPS community (56% of the bootstrap samples) as to the DWB-M community (44%). Otherwise, the four-dimensional structure of the network was stable (figure 3*c*): the same communities reliably emerged across all 1000 iterations and the four-dimensional structure of the network was replicated 100% of the time. The centrality indices (figure 3*d*) suggest that there were no items with a particular strong influence on the network, only Item 8 of the SBPS had an elevated influence that might reflect its intermediary role between boredom proneness and maladaptive ways of DWB.

### 4.3. Predicting adaptive and maladaptive behaviour

An EGA revealed that the 19 items assessing adaptive and maladaptive behaviours belonged to four communities: one comprising the adaptive behaviours (e.g. physical activity) and the other three comprising the maladaptive behaviours. These latter communities pertained to excessive media use (e.g. watching TV), risky behaviour (e.g. fast and reckless driving) and harmful behaviour (e.g. problematic eating), respectively. Accordingly, we obtained four network scores (i.e. one per community) for each participant. The network scores for maladaptive behaviours were significantly correlated with boredom proneness (0.45, 95% CI [0.38, 0.51] ≤ *r* ≤ 0.53, 95% CI [0.47, 0.58]; 0.40, 95% CI [0.33, 0.48] ≤ *r*_boot_ ≤ 0.49, 95% CI [0.43, 0.55]), boredom avoidance and escape tendencies (0.19, 95% CI [0.11, 0.26] ≤ *r* ≤ 0.21, 95% CI [0.13, 0.28]; 0.11, 95% CI [0.03, 0.18] ≤ *r*_boot_ ≤ 0.19, 95% CI [0.11, 0.26]), adaptive ways of DWB (−0.18, 95% CI [−0.26, −0.11] ≤ *r* ≤ −0.16, 95% CI [−0.23, −0.08]; −0.27, 95% CI [−0.34, −0.18] ≤ *r*_boot_ ≤ −0.17, 95% CI [−0.26, −0.09]), and maladaptive ways of DWB (0.46, 95% CI [0.40, 0.52] ≤ *r* ≤ 0.57, 95% CI [0.52, 0.62]; 0.43, 95% CI [0.37, 0.51] ≤ *r*_boot_ ≤ 0.55, 95% CI [0.49, 0.61]). The network score for adaptive behaviour was significantly correlated with boredom proneness (*r* = −0.44, 95% CI [−0.50, −0.38], *r*_boot_ = −0.45, 95% CI [−0.52, −0.38]) as well as with adaptive (*r* = 0.39, 95% CI [0.32, 0.46], *r*_boot_ = 0.39, 95% CI [0.31, 0.46]) and maladaptive ways of DWB (*r* = −0.29, 95% CI [−0.36, −0.22], *r*_boot_ = −0.31, 95% CI [−0.38, −0.23]), whereas no significant correlation with boredom avoidance and escape tendencies emerged (*r* = 0.06, 95% CI [−0.02, 0.14], *r*_boot_ = 0.08, 95% CI [−0.01, 0.16]). To scrutinize the unique contributions of the boredom-related scales in explaining adaptive and maladaptive behaviours, we conducted several regression analyses with the network scores as dependent variables (table 4).

**Table 4.** Regression of network scores representing adaptive and maladaptive behaviours on the SBPS, BAE and DWB scales.

	adaptive behaviour			excessive media use			risky behaviour			harmful behaviour		
	*b*	*b*_boot_	*β*	*b*	*b*_boot_	*β*	*b*	*b*_boot_	*β*	*b*	*b*_boot_	*β*
***Model 1***	*R*² = 0.20***			*R*² = 0.20***			*R*² = 0.23***			*R*² = 0.28***		
Intercept	6.24*** [6.08, 6.41]	6.24*** [6.07, 6.41]		1.87*** [1.69, 2.05]	1.87*** [1.67, 2.07]		1.00*** [0.86, 1.14]	1.00*** [0.84, 1.14]		1.07*** [0.95, 1.19]	1.07*** [0.94, 1.19]	
SBPS	−0.33*** [−0.38, −0.28]	−0.33*** [−0.39, −0.27]	−0.44*** [−0.51, −0.37]	0.37*** [0.31, 0.43]	0.37*** [0.30, 0.44]	0.45*** [0.38, 0.52]	0.30*** [0.26, 0.35]	0.30*** [0.25, 0.37]	0.48*** [0.41, 0.55]	0.30*** [0.26, 0.34]	0.30*** [0.25, 0.35]	0.53*** [0.46, 0.59]
***Model 2***	*R*² = 0.22***			*R*² = 0.22***			*R*² = 0.24***			*R*² = 0.28***		
Intercept	5.90*** [5.67, 6.13]	5.90*** [5.66, 6.13]		1.52*** [1.27, 1.78]	1.53*** [1.27, 1.79]		0.75*** [0.56, 0.95]	0.75*** [0.51, 0.97]		0.90*** [0.73, 1.07]	0.90*** [0.68, 1.10]	
SBPS	−0.35*** [−0.40, −0.30]	−0.35*** [5.66, 6.13]	−0.47*** [−0.54, −0.40]	0.35*** [0.29, 0.41]	0.35*** [0.28, 0.41]	0.42*** [0.35, 0.49]	0.29*** [0.25, 0.33]	0.29*** [0.23, 0.35]	0.45*** [0.39, 0.52]	0.29*** [0.25, 0.33]	0.29*** [0.24, 0.34]	0.51*** [0.44, 0.57]
BAE	0.10*** [0.05, 0.14]	0.10*** [−0.41, −0.29]	0.15*** [0.08, 0.22]	0.10*** [0.05, 0.15]	0.10*** [0.04, 0.15]	0.13*** [0.06, 0.20]	0.07*** [0.03, 0.11]	0.07*** [0.03, 0.11]	0.12*** [0.05, 0.19]	0.05** [0.01, 0.08]	0.05** [0.02, 0.08]	0.09** [0.03, 0.16]
***Model 3***	*R*² = 0.26***			*R*² = 0.26***			*R*² = 0.34***			*R*² = 0.38***		
Intercept	4.80*** [4.38, 5.22]	4.79*** [4.31, 5.28]		1.55*** [1.36, 1.74]	1.55*** [1.33, 1.76]		0.66*** [0.52, 0.80]	0.66*** [0.48, 0.82]		0.77*** [0.65, 0.90]	0.77*** [0.61, 0.92]	
SBPS	−0.26*** [−0.31, −0.20]	−0.26*** [−0.32, −0.19]	−0.35*** [−0.42, −0.28]	0.22*** [0.15, 0.29]	0.22*** [0.14, 0.30]	0.27*** [0.19, 0.35]	0.15*** [0.10, 0.20]	0.15*** [0.09, 0.20]	0.23*** [0.15, 0.31]	0.16*** [0.12, 0.21]	0.16*** [0.12, 0.21]	0.29*** [0.21, 0.37]
DWB-A	0.23*** [0.17, 0.30]	0.23*** [0.16, 0.30]	0.27*** [0.20, 0.34]									
DWB-M				0.25*** [0.18, 0.32]	0.25*** [0.18, 0.33]	0.31*** [0.22, 0.39]	0.26*** [0.21, 0.31])	0.26*** [0.21, 0.32]	0.42*** [0.34, 0.49]	0.23*** [0.18, 0.27]	0.23*** [0.18, 0.27]	0.40*** [0.32, 0.48]
***Model 4***	*R*² = 0.26***			*R*² = 0.27***			*R*² = 0.35***			*R*² = 0.38***		
Intercept	4.74*** [4.32, 5.16]	4.74*** [4.25, 5.22]		1.30*** [1.04, 1.55]	1.30*** [1.04, 1.55]		0.51*** [0.32, 0.69]	0.50*** [0.29, 0.72]		0.68*** [0.52, 0.85]	0.68*** [0.48, 0.89]	
SBPS	−0.28*** [−0.33, −0.22]	−0.28*** [−0.34, −0.21]	−0.37*** [−0.44, −0.30]	0.21*** [0.14, 0.28]	0.21*** [0.13, 0.29]	0.26*** [0.18, 0.34]	0.14*** [0.09, 0.19]	0.14*** [0.09, 0.20]	0.23*** [0.15, 0.30]	0.16*** [0.12, 0.20]	0.16*** [0.12, 0.21]	0.28*** [0.21, 0.36]
BAE	0.05* [0.01, 0.10]	0.05* [0.01, 0.10]	0.08* [0.01, 0.15]	0.08** [0.03, 0.13]	0.08* [0.02, 0.13]	0.10** [0.04, 0.17]	0.05* [0.01, 0.08]	0.05* [0.01, 0.08]	0.08* [0.02, 0.15]	0.03 [0.00, 0.06]	0.03 [0.00, 0.06]	0.05 [−0.01, 0.12]
DWB-A	0.21*** [0.15, 0.28]	0.21*** [0.14, 0.29]	0.24*** [0.17, 0.32]									
DWB-M				0.24*** [0.17, 0.30]	0.24*** [0.16, 0.31]	0.29*** [0.21, 0.37]	0.26*** [0.21, 0.30]	0.26*** [0.20, 0.31]	0.40*** [0.32, 0.48]	0.22*** [0.18, 0.26]	0.22*** [0.18, 0.27]	0.39*** [0.32, 0.47]

*Note. b,* unstandardized coefficients; *b*_boot_, bootstrapped coefficients; *β*, standardized coefficients.

***p < 0.001, **p < 0.01, *p < 0.05.

Higher boredom proneness (SBPS) predicted less adaptive behaviour as well as more excessive media usage, more risky behaviour and more harmful behaviour (Model 1), |*b*| ≥ 0.30, s.e. ≤ 0.02, *p* < 0.001. Beyond these effects, the urge to avoid and escape boredom (BAE) predicted *more* adaptive behaviour as well as more excessive media usage, more risky behaviour, and more harmful behaviour (Model 2), *b* ≥ 0.05, s.e. ≤ 0.02, *p* ≤ 0.006. Adaptive ways of dealing with boredom (DWB-A) predicted more adaptive behaviour beyond the effects of the SBPS, *b* = 0.23, s.e. = 0.03, *p* < 0.001, while maladaptive ways of dealing with boredom (DWB-M) predicted more excessive media usage, more risky behaviour, and more harmful behaviour, *b* ≥ 0.23, s.e. ≤ 0.02, *p* < 0.001 (Model 3). Finally, all scales made significant individual contributions to the prediction of adaptive and maladaptive behaviour (Model 4), with the sole exception that BAE did not predict harmful behaviour beyond SBPS and DWB-M, *b* = 0.03, s.e. = 0.02, *p* = 0.098.

## 5. Discussion

Functional accounts of boredom propose that state boredom is an impartial signal to change something about one's current situation, which might spur adaptive and maladaptive behaviour alike. This prediction seems to be at odds with the bulk of research that has linked individual differences in boredom proneness to less adaptive behaviours and to more maladaptive behaviours. However, this apparent discrepancy probably reflects that the concept of boredom proneness captures an individual's inability to lead a life that is not boring, while it is agnostic to an individual's intrinsic tendency to experience an urge to avoid or escape boredom in the first place. Crucially, this urge lies at the heart of the definition of boredom [4] and functional accounts of boredom [14,15,22]. We addressed this issue by disentangling individual differences in boredom proneness from individual differences in tendencies to avoid, escape from and deal with boredom. Our findings replicated the well-established association between boredom proneness and less adaptive and more maladaptive behaviour. Crucially, and in line with predictions derived from functional accounts of boredom, people with a greater tendency to avoid and escape boredom were more likely to deal with boredom in both adaptive and maladaptive ways and reported to engage in more adaptive and more maladaptive behaviour alike.

Our observation that boredom is linked to adaptive and maladaptive behaviour alike shows parallels to research on coping with boredom at school [74], which has identified sets of both adaptive (e.g. reminding themselves of the importance of the subject) and maladaptive strategies (e.g. talking to classmates) that students apply to deal with boredom. However, there are important differences between our present research and research on coping strategies. First, the boredom coping strategies investigated by Nett *et al*. [74] were derived from models of coping with stress, and it is unclear whether these models capture the scope of behaviours people may use to deal with boredom. By contrast, the DWB scale does not make assumptions about the specific strategies people might use. Second, the strategies assessed by Nett *et al*. [74] are not designed to measure directly whether people deal with boredom in adaptive versus maladaptive ways. Instead, the adaptiveness of the identified strategies is determined ex post by correlating them with various outcomes. Whether a specific strategy is adaptive or maladaptive thus depends on the choice of these outcomes. In the DWB, adaptiveness can be deduced directly from participant's answers and thus provides a more direct assessment of the construct of interest. Third, the coping strategies identified by Nett *et al*. [74] have so far been evaluated in the academic context alone, which differs from other contexts in several crucial ways. Most importantly, school settings heavily restrict the behaviours students can and will perform (e.g. by sanctioning maladaptive behaviours). Despite these differences, both lines of research arrive at similar conclusions, and this highlights the relevance of capturing the responses to boredom in a standardized manner. In this vein, it would be an intriguing research question to examine how the narrower conceptualization of student-specific coping strategies maps onto the assessment of adaptive and maladaptive behaviours that are assessed on a more general level with the DWB scale.

An essential insight is that boredom proneness is not what one would understand as *trait boredom*. The concept of boredom proneness is tightly linked to the boredom proneness scale [11], which has been developed at a time when there was no generally accepted definition or theory of boredom. Therefore, it is not surprising that the concept of boredom proneness is somewhat fuzzy and shows little overlap with the contemporary understanding of boredom [23]. For instance, it has been suggested that boredom proneness overlaps to a substantial degree with poor self-control. This is indicated by sizeable correlations between the SBPS and measures of self-control [29,31,51] and their overlapping content domains in psychometric network analyses [50]. In fact, SBPS scores and traditional measures of trait self-control have even been found to map onto the same adaptive (low SBPS, high self-control) and less adaptive (high SBPS, low self-control) latent personality profiles [29]. Accordingly, the well-established links between poor self-control and less adaptive/more maladaptive behaviours (e.g. [52–54]) might explain the sizeable associations between these behaviours and the SBPS especially in comparison with the BAE scale, as well as their robustness to adjusting for BAE scores. By developing a measure of the tendency to avoid and escape boredom, we made a crucial step towards developing measures of individual differences in boredom that are better aligned with the definition and theories of boredom. Researchers interested in measuring boredom as a trait consistent with how boredom is conceptualized (rather than the negative consequences of consistently DWB) might therefore use the BAE scale rather than (or in addition to) the boredom proneness scale in future studies.

Another intriguing finding was that boredom avoidance and escape tendencies were significantly related with the engagement in adaptive behaviours only when boredom proneness was controlled for in the regression analyses (i.e. non-significant bivariate correlation). This points to a possible asymmetry between adaptive and maladaptive behaviour: the former might require more self-control than the latter (for the importance of self-control for adaptive behaviour, see [52]). Even though boredom might be impartial with regard to the adaptivity of behaviour, it could thus still be more likely to result in maladaptive behaviours merely because these might require less effort and could be more readily available than adaptive behaviours. Accordingly, adjusting for self-control should be the better test of the theoretically predicted association between avoidance and escape tendencies and adaptive behaviours. And indeed, due to the overlap between measures of boredom proneness and measures of self-control [50], adjusting for boredom proneness in the regression analyses might have partialled out the common variance of boredom proneness and self-control. That being said, measures of boredom proneness and self-control cannot be equated (e.g. shared variance might reflect a common underlying construct), and directly controlling for self-control is thus necessary to evaluate this idea. A promising route of future research therefore is to add measures of self-control when investigating the association between boredom avoidance and escape tendencies and adaptive behaviours. Unlike boredom proneness, boredom avoidance and escape tendencies should display only weak associations with measures of self-control.

Boredom is understood to trigger overt behavioural responses that might (for maladaptive responses) or might not (for adaptive responses) require the application of self-control to regulate goal-directed behaviour. However, boredom has also been associated with covert behavioural responses. More specifically, boredom has been linked to daydreaming and mind-wandering. For instance, prior research has shown that boredom proneness is linked to trait measures of mind-wandering [75]. Interestingly, this relationship is much stronger for spontaneous mind-wandering than for deliberate mind-wandering. The former refers to the tendency that one's thoughts drift off in an uncontrolled fashion (example item of the spontaneous mind-wandering scale: ‘It feels like I don't have control over when my mind wanders' [76], whereas deliberate mind-wandering reflects a more strategic and controlled way of attending inner worlds (example item of the deliberate mind-wandering scale: ‘I find mind-wandering is a good way to cope with boredom' [76]. Thus, mind-wandering in boredom-prone people seems to be less under their volitional control, again attesting to the notion of boredom proneness as being a rather maladaptive self-regulatory disposition where one is unable to regulate one's interaction with the—internal or external—environment in a way that is conducive to adaptive outcomes. Similarly to the reasoning behind disentangling the link between boredom and self-control, one might expect a markedly different relationship between boredom as measured by boredom avoidance and escape scale and mind-wandering. For example, given that deliberate mind-wandering probably is an adaptive response to boredom when other options are exhausted, one might expect stronger relationships between this type of mind-wandering and the newly developed boredom avoidance and escape scale. Likewise, as spontaneous mind-wandering essentially reflects a lack of agency over the directions of one's own thoughts, one might expect a lower correlation between this type of mind-wandering and the active responses people high in boredom avoidance and escape scale make in order to avoid being bored.

Our study has limitations that should be taken into account when interpreting the findings. First, our research is based on self-reported behaviour, which might have biased our findings. For instance, there might be a discrepancy between how people report to deal with boredom and how they actually deal with boredom. Therefore, we consider it an important next step to validate the BAS and the DWB scales with objective assessments of behaviour in boring situations. Second, our assessment of adaptive and maladaptive behaviours relied on participants' evaluation on the (mal-)adaptivity of their behaviour. As research on boredom coping strategies suggests (e.g. [74]), an alternative is to measure specific behavioural and cognitive strategies that can be classified in terms of adaptivity. Accordingly, it would be interesting to investigate the associations between the DWB scale and measures of specific boredom coping strategies. Third, we focused solely on measuring boredom and did not include measures of related constructs, such as self-control or mind-wandering. Exploring how these constructs contribute to the nomological network spanned by the different measures of boredom would shed light on the construct of boredom itself.

## Data Availability

All data, code and materials used in this research are available at OSF (https://osf.io/nbw95/).

## Footnotes

1 Further attesting to the complexity of the construct, other forms of boredom have been identified in the literature (e.g. profound boredom [1]).
